# The anti-obesity effects of a tuna peptide on 3T3-L1 adipocytes are mediated by the inhibition of the expression of lipogenic and adipogenic genes and by the activation of the Wnt/β-catenin signaling pathway

**DOI:** 10.3892/ijmm.2015.2231

**Published:** 2015-06-03

**Authors:** YOUNG-MIN KIM, IN-HYE KIM, JEONG-WOOK CHOI, MIN-KYEONG LEE, TAEK-JEONG NAM

**Affiliations:** Department of Food Science and Nutrition, Pukyong National University, Busan 608-737, Republic of Korea

**Keywords:** CCAAT/enhancer-binding proteins, Wnt-10b, adipon-ectin, 3T3-L1 cells, tuna peptide

## Abstract

The differentiation of 3T3-L1 cells into adipocytes involves the activation of an organized system of obesity-related genes, of which those encoding CCAAT/enhancer-binding proteins (C/EBPs) and the Wnt-10b protein may play integral roles. In a previous study of ours, we found that a specific peptide found in tuna (sequence D-I-V-D-K-I-E-I; termed TP-D) inhibited 3T3-L1 cell differentiation. In the present study, we observed that the expression of expression of C/EBPs and Wnt-10b was associated with obesity. The initial step of 3T3-L1 cell differentiation involved the upregulation of C/EBP-α expression, which in turn activated various subfactors. An upstream effector of glycogen synthase kinase-3β (GSK-3β) inhibited Wnt-10b expression in 3T3-L1 adipocytes. In a previous study of ours, we sequenced the tuna peptide via sodium dodecyl sulfate-polyacrylamide gel electrophoresis (SDS-PAGE) and quadrupole time-of-flight mass spectrometry (Q-TOF MS/MS) and confirmed the anti-obesity effects thereof in 3T3-L1 adipocytes. In the present study, we demonstrate that TP-D inhibits C/EBP and promotes Wnt-10b mRNA expression, thus activating the Wnt pathway. The inhibition of lipid accumulation was measured using a glucose and triglyceride (TG) assay. Our results confirmed that TP-D altered the expression levels of C/EBP-related genes in a dose-dependent manner and activated the Wnt signaling pathway. In addition, we confirmed that total adiponectin and high-molecular weight (HMW) adiponectin levels were reduced by treatment with TP-D. These data indicate that TP-D inhibits adipocyte differentiation through the inhibition of C/EBP genes and the subsequent activation of the Wnt/β-catenin signaling pathway.

## Introduction

Obesity has a partly genetic basis; genes of the endocrine system and metabolism may favor obesity. However, the direct cause of obesity is an imbalance between energy storage and consumption, and thus an imbalance between calorie intake and consumption (1,2). The incidence and severity of obesity-related diseases, including circulatory diseases, such as diabetes, cancer and high blood pressure render obesity one of the leading causes of mortality (3,4). Physiologically, obesity is associated with increased levels of fat within adipocytes and/or an increase in the number of adipocytes. Accumulated intracellular triglycerides (TGs) can be broken down by exercise or diet; however, obesity caused by increased numbers of adipocytes is difficult to treat as the fat cells must be destroyed or removed. The differentiation of pre-adipocytes into adipocytes may be triggered by changes in specific hormone levels and/or excessive nutritional intake during the growth process. Adipocyte differentiation may occur in particular regions of the body. In addition, due to limitations in the size of adipocytes, the number of such cells increases with excess energy consumption, in order to provide storage for these excess energy levels (5). The 3T3-L1 cell line is a pre-adipocyte line derived from mouse embryonic fibroblasts and is the most commonly used line in obesity research.

In the case that glucose and TGs accumulate and the CCAAT/enhancer-binding protein (C/EBP)-β or -δ is induced during early adipocyte differentiation, the activation of C/EBP-α, a peroxisome proliferator-activated receptor (PPAR) transcription factor, occurs late in differentiation, in turn causing the transcriptional activation of various adipocyte-specific mRNAs encoding fatty acid synthase (FAS), lipoprotein lipase (LPL), acetyl-CoA carboxylase (ACC), stearoyl-CoA desaturase-1 (SCD-1) and PPAR co-activator-1 (PGC-1). These enzymes are involved in lipogenesis and adipogenesis, and induce the synthesis of fat globules. Wnts are a family of secreted glycoproteins with a high number of conserved cysteines. They regulate various morphogenetic processes by controlling the expression of a number of genes through several steps triggered when Wnt receptors are activated by binding to the receptors in surrounding cells (6–8). The Wnt canonical signal transmission system plays a major role in the differentiation of adipocytes. As previously demonstrated, when a canonical signal transmission system, such as Wnt is activated in pre-adipocytes and Wnt-10b is overexpressed, adipocyte formation is prevented through the inhibition of C/EBPs and PPAR-γ (key factors in adipocyte cell differentiation), in turn suppressing the expression of adipocyte-specific genes (9). In addition, the balance between β-catenin and PPAR-γ levels is important in terms of adipocyte differentiation; maintaining this equilibrium depends on proteolysis following the phosphorylation of β-catenin by glycogen synthase kinase-3β (GSK-3β) (10).

Tuna is known to have anti-arteriosclerotic and anti-obesity properties, and to reduce the levels of cholesterol in the blood. Processed tuna is principally muscle tissue. In a previous study of ours, we analyzed boiled tuna extract, identified a relevant peptide in that extract, synthesized that peptide (11), and measured the anti-obesity effects thereof. We found that the peptide inhibited the adipocyte differentiation of 3T3-L1 cells.

## Materials and methods

### Preparation of desalinated boiled tuna extract

The desalinated boiled tuna extract used in this study was prepared in 2014 in Korea. First, the boiled tuna extract was centrifuged to remove suspended solids that may interfere with desalination. This process changed the Brix status from 55 to 45 Brix, and the salinity from 12 to 13%. Membrane filtration was performed (cut-off, 200 Da). We obtained a solution of 30 Brix and 1% salinity and sterilized the material using a heat exchanger (conditions: 110°C for 10 sec). The sample was then placed in a 1.5-ml tube and stored at −70°C until use.

### Preparation of soluble/insoluble tuna protein and synthesis of the tuna peptide

The desalinated boiled tuna extract was incubated in a Tween-20 extraction buffer overnight at room temperature, followed by centrifugation at 5,000 rpm for 10 min at 4°C. The upper phase was mixed with cold methanol/chloroform to induce protein separation. The solution was then centrifuged at 12,000 rpm for 5 min at 4°C. The aqueous layer was removed, and cold methanol was added, followed by centifugation at 12,000 rpm for 10 min at 4°C. The supernatant was removed and dried.

The molecular weights of the tuna proteins were investigated by Coomassie blue (7% acetic acid, 40% methanol, and 0.1% bromophenol blue; all v/v) staining of SDS-PAGE gels and destaining (7% acetic acid, 20% methanol; both v/v). Tuna proteins approximately 10 kDa in size were analyzed by quadrupole-time-of-flight tandem mass spectrometry (Q-TOF MS/MS). The peptide mixture was desalinated and concentrated in a C18 column packed with POROS R2 (20–30 *µ*m bead size; PerSeptive Biosystems, Inc., Framingham, MA, USA). MS/MS involved nano-electrospray ionization (ESI) and micro Q-TOF MS (Bruker Daltonics, Bremen, Germany). The tuna-derived peptide D-I-V-D-K-I-E-I (TP-D) was synthesized by Peptron (Daejeon, Korea), and the purified tuna peptide was synthesized on a C18 column (Shiseido Capcell Pak; Shiseido, Tokyo, Japan) using a Shimadzu Prominence HPLC system (Shimadzu, Kyoto, Japan). The eluent was placed in 0.1% (v/v) trifluoroacetic acid (TFA)/water with a gradient of 0–90% (v/v) acetonitrile. The flow rate was 1 ml/min, and detection was carried out via UV at 220 nm. The molecular weight of TP-D was 944 Da (determined using an HP 1100 Series LC/MSD system; Agilent Technologies, Santa Clara, CA, USA).

### Cell culture and adipocyte differentiation

3T3-L1 mouse pre-adipocytes (derived from fibroblasts; American Type Culture Collection, Manassas, VA, USA) were maintained at a temperature of 37°C in a humidified atmosphere containing 5% (v/v) CO_2_. The cells were cultured in Dulbecco’s modified Eagle’s medium (DMEM) supplemented with 10% (v/v) bovine calf serum (BCS;HyClone, Logan, UT, USA) and penicillin/streptomycin (100 U/ml/100 mg/ml). The cells were cultured to 60–80% confluence in a 6-well plate and, upon reaching confluence, were allowed to grow for an additional 2–4 days in DMEM with 10% (v/v) fetal bovine serum (FBS; HyClone). Cell differentiation was initiated by treatment with MDI [0.5 mM 3-isobutyl-1-methylxanthine (IBMX), 0.25 *µ*M dexamethasone and 10 mg/l insulin] for 48 h. The medium was then replaced with DMEM containing 10 mg/l insulin and changed every 2 days.

### Cell proliferation assays

The 3T3-L1 cell proliferation was measured using the CellTiter 96^®^ AQueous Non-Radioactive Cell Proliferation assay (Promega Corp., Madison, WI, USA). The assay is based on the cleavage of 3-(4,5-dimethylthiazol-2-yl)-5-(3-carboxymethoxy-phenyl)-2-(4-sulfonyl)-2H-tetra-zolium (MTS) to a formazan product soluble in the cell culture medium. The cells were seeded into 96-well plates at 2×10^4^ cells/well in 100 *µ*l of medium and maintained for 24 h; the medium was then replaced with serum-free medium (SFM). After 24 h, the medium was replaced with SFM containing TP-D (125, 250, 500 or 1,000 *µ*g/ml) for 24 h. Subsequently, the cells were incubated with MTS solution for 30 min at 37°C. Cell proliferation was determined by measuring the absorbance at 490 nm using a Benchmark enzyme-linked immunosorbent assay (ELISA) plate reader (Bio-Rad Laboratories, Hercules, CA, USA).

### Glucose uptake assay

The 3T3-L1 pre-adipocytes were incubated in DMEM containing 10% (v/v) BCS. Cell differentiation was induced by treatment with MDI in fresh DMEM containing 10% (v/v) FBS. Following differentiation, the medium was replaced with SFM containing TP-D at concentrations of 125, 250, 500 or 1,000 ng/ml for 48 h prior to the glucose uptake assay using a glucose kit protocol (Asan Pharmaceutical Co., Ltd., Hwaseong, Korea). The test enzyme was added to the culture medium maintained at 37°C for 5 min in a humidified atmosphere containing 5% (v/v) CO_2_. The absorbance at 500 nm was measured within 40 min.

### TG assay

The 3T3-L1 pre-adipocytes were incubated in DMEM containing 10% (v/v) BCS. Cell differentiation was induced by treatment with MDI in fresh DMEM with 10% (v/v) FBS. Following the induction of cell differentiation, the medium was replaced with SFM containing TP-D at concentrations of 125, 250, 500 and 1,000 ng/ml for 48 h prior to the TG assay. Cell pellets were ruptured with phosphate-buffered saline (PBS), and the TG levels were assayed using a TG kit protocol (obtained from Asan Pharmaceutical Co., Ltd.). The test enzyme (TG-measured solution) was added to the lysate supernatants, and the cells were maintained at 37°C for 5 min in a humidified atmosphere containing 5% CO_2_. The absorbance at 550 nm was measured within 60 min.

### mRNA expression by RT-PCR

The 3T3-L1 pre-adipocytes were seeded into 6-well plates at 2×10^4^ cells/well in 2 ml of medium. Cell differentiation was then induced by treatment with MDI, as described in the experiments above (11). Following the induction of cell differentiation, the medium was replaced with SFM containing TP-D (500 or 1,000 ng/ml) for 48 h and the cells were then treated with 1 ml of TRIzol reagent (Invitrogen Life Technologies, Carlsbad, CA, USA). Subsequently, the samples were centrifuged at 14,000 rpm for 15 min at 4°C, after adding 200 *µ*l of chloroform. The samples were divided into supernatant and pellet (0.1% of DEPC was added to 50 *µ*l of water). The mRNA levels in the cell supernatants were quantified using an Oligo(dT) primer (Intron Biotechnology, Seoul, Korea) and the cDNA was synthesized at 50°C for 1 h, 95°C for 5 min. The cDNA was added to 2X TOPsimple™ DyeMIX-nTaq (Enzynomics, Daejeon, Korea), and primers were added to 0.1% (v/v) diethylpyrocarbonate (DEPC) in water. The PCR reactions for the amplification of the DNA were run on 1% (w/v) agarose gels and the nuclei were stained using RedSafe Nucleic Acid Staining solution (Intron Biotechnology). Initial denaturation was performed at 95°C for 2 min and the denaturation step at 95°C for 30 sec. In order to allow annealing of the primers to the single-stranded DNA template, the temperature was lowered to 50–55°C for 30 sec. The elongation step was performed at 72°C for 1 min and the final elongation at 72°C for 5 min.

### Western blot analysis

The 3T3-L1 pre-adipocytes were seeded into 6-well plates at 2×10^4^ cells/well in 2 ml of medium. Cell differentiation was induced by treatment with MDI, as described above (11). Following differentiation, the medium was replaced with SFM containing TP-D (500 or 1,000 ng/ml) for 48 h. The cells were then washed with PBS, and lysis buffer was added [20 mM Tris Base (pH 8), 150 mM NaCl, 100 *µ*M sodium vanadate, 100 *µ*M ammonium molybdate, 10% (v/v) glycerol, 0.1% (v/v) Nonidet P-40, 0.1% (w/v) SDS, 1 mM glycerophos-phate, 1 *µ*g/ml aprotinin, 1 *µ*g/ml leupeptin, 1 *µ*g/ml pepstatin A and 1 mM phenylmethanesulfonyl fluoride (PMSF)]. Proteins were separated by 7–15% (w/v) SDS-PAGE and transferred onto polyvinylidene fluoride membranes (Millipore, Billerica, MA, USA), which were blocked at room temperature with 1% (w/v) bovine serum albumin in TBS-T [10 mM Tris-HCl (pH 7.5), 150 mM NaCl, 0.1% (v/v) Tween-20] and then incubated with the following antibodies while being shaken: anti-C/EBP-α (sc-9314, anti-rabbit; 1:1,000), anti-C/EBP-β (sc-150, anti-rabbit; 1:1,000), anti-C/EBP-δ (sc-151, anti-rabbit; 1:1,000), anti-PPAR-γ (sc-1984, anti-goat; 1:1,000), anti-CD36 (sc-7641, anti-goat; 1:1,000), anti-sterol regulatory element-binding protein 1 (SREBP-1; sc-366, anti-rabbit; 1:1,000), anti-FAS (sc-7886, anti-mouse; 1:1,000), anti-ACC (sc-271965, anti-mouse; 1:1,000), anti-LPL (sc-32382, anti-goat; 1:1,000), anti-suppressor of cytokine signaling-3 (SOCS-3; sc-73045, anti-mouse; 1:1,000), anti-fatty acid binding protein (FABP; sc-18661, anti-goat; 1:1,000), anti-uncoupling protein (UCP)-1 (sc-6529, anti-goat; 1:1,000), anti-UCP-2 (sc-6525, anti-goat; 1:1,000), anti-glucose transporter type 4 (GLUT4; sc-1606, anti-goat; 1:1,000), anti-Wnt-10b (sc-25524, anti-rabbit; 1:1,000), anti-Frizzled (sc-130758, anti-rabbit; 1:1,000), anti-lipoprotein receptor-related protein-6 (LRP6; sc-25317, anti-mouse; 1:1,000), anti-dishevelled (Dvl; sc-166303, anti-mouse; 1:1,000), anti-β-catenin (sc-1496, anti-goat; 1:1,000), anti-GSK-3β (sc-377213, anti-mouse; 1:1,000), anti-T cell factor (TCF; sc-271453, anti-mouse; 1:1,000), anti-lymphoid enhancer-binding factor (LEF; sc-28687, anti-rabbit; 1:1,000), anti-cyclin D1 (CCND1; sc-753, anti-rabbit; 1:1,000) or anti-glyceraldehyde 3-phosphate dehydrogenase (GAPDH; sc-25778, anti-rabbit; 1:1,000) antibodies (all from Santa Cruz Biotechnology, Inc., Santa Cruz, CA, USA). The secondary antibodies were peroxidase-conjugated goat (sc-2741), mouse (sc-2032) or rabbit (sc-2031) antibody (1:10,000; GE Healthcare Bio-Sciences, Piscataway, NJ, USA). Proteins were visualized by exposure to SuperSignal West Pico Stable Peroxide solution and SuperSignal West Pico Luminol/Enhancer solution (both from Thermo Fisher Scientific, Rockford, IL, USA) and Kodak X-ray film.

### Assays of total and high-molecular weight (HMW) adiponectins

Adiponectin and HMW adiponectin levels were measured using an ELISA kit (ALPCO Diagnostics, Salem, NH, USA). The 3T3-L1 pre-adipocytes were incubated with DMEM containing 10% (v/v) BCS in a 6-well plate. Cell differentiation was induced by treatment with MDI, as described above (11). Following differentiation, the medium was replaced with SFM containing TP-D (500 or 1,000 ng/ml) for 48 h. A total of 50 *µ*l of medium was assayed. The culture medium and cell lysate were incubated with 100 *µ*l of protease buffer, which was then neutralized with 100 *µ*l of pre-treatment buffer. The absorbances of total and HMW adiponectin at 492 nm were measured.

### Statistical analysis

The results are presented as the means ± SD and were analyzed using SPSS version 10.0 software (SPSS,Inc., Chicago, IL, USA). Data were validated by ANOVA, and a P-value <0.05 as indicated by Duncan’s multiple range test was considered to indicate a statistically significant difference.

## Results

### TP-D is not toxic to 3T3-L1 pre-adipocytes

**Figure 1:**
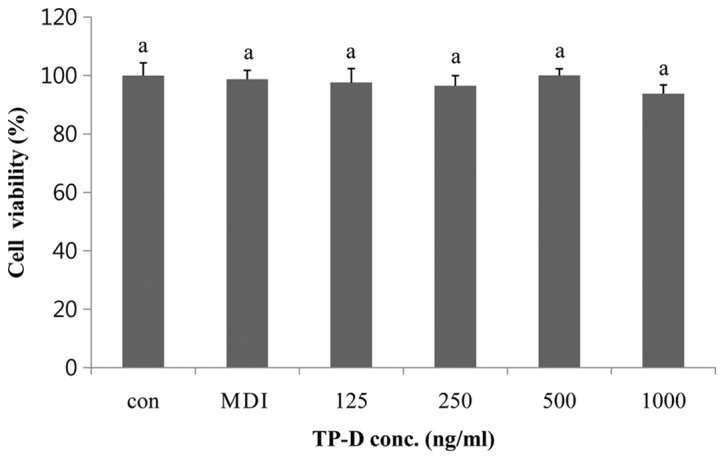
Effect of the tuna-derived peptide, D-I-V-D-K-I-E-I (TP-D), on the viability of 3T3-L1 pre-adipocytes. The 3T3-L1 cells were treated with various concentrations of TP-D (125, 250, 500 or 1,000 ng/ml) for 48 h. Cell viability was measured at 490 nm using by MTS assay. Values represent the means ± SD; P<0.05 as shown by ANOVA. Bars labeled with different letters indicate significant differences according to Duncan’s multiple range test. Bars labeled with the same letter indicate no significant difference according to Duncan’s multiple range test. Con, control (undifferentiated cells); conc., concentration.

The effect of TP-D on the viability of the 3T3-L1 pre-adipocytes was investigated by MTS assay. The cells were seeded into 96-well plates at 2×10^4^ cells/well in 100 *µ*l of medium and allowed to attach for 24 h. The medium was replaced with SFM for 4 h, followed by treatment of the 3T3-L1 cells with various concentrations of TP-D (125, 250, 500 and 1,000 *µ*g/ml) for 24 h. MTS solution was then added and cell viability was measured. TP-D was not found to be toxic to the cells, as no significant decrease in cell viability was observed at any of the concentrations used (Fig. 1).

### Effect of TP-D on glucose uptake by 3T3-L1 cells

**Figure 2:**
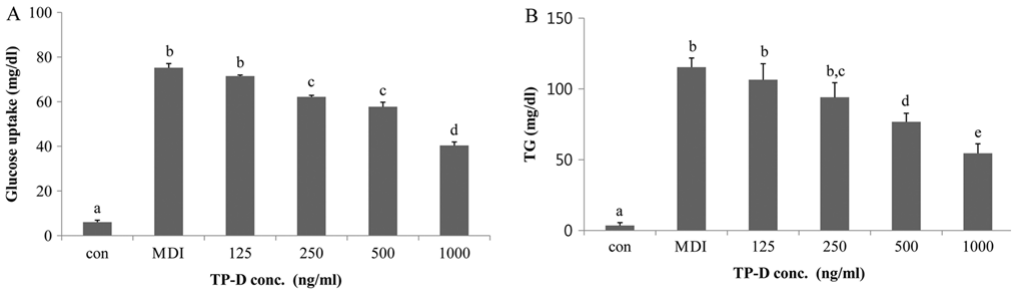
Effect of the tuna-derived peptide, D-I-V-D-K-I-E-I (TP-D), on glucose uptake and triglyceride (TG) levels in differentiated 3T3-L1 adipocytes. (A) The 3T3-L1 cells were treated with TP-D at various concentrations (125, 250, 500 or 1,000 ng/ml) for 48 h. Glucose levels in the culture medium were measured by a glucose uptake assay using an ELISA reader. (B) The 3T3-L1 cells were treated with TP-D at various concentrations (125, 250, 500 or 1,000 ng/ml) for 48 h. TG levels in the culture medium were measured by a TG assay using an ELISA reader. Values represent the means ± SD; P<0.05 as shown by ANOVA. Bars labeled with different letters indicate significant differences according to Duncan’s multiple range test. Con, control (undifferentiated cells); conc., concentration.

Glucose consumption is a prerequisite for 3T3-L1 cell differentiation. We compared glucose consumptions of differentiated and undifferentiated cells treated with TP-D. The cells were incubated with TP-D at concentrations of 125, 250, 500 and 1,000 ng/ml for 48 h. In the TP-D-treated group, glucose uptake decreased compared with the MDI-reated group. Glucose uptake was significantly decreased with all the tested concentrations of TP-D, indicating that TP-D inhibited glucose consumption and thus the differentiation of 3T3-L1 cells (Fig. 2A).

### Effects of TP-D on TG levels in 3T3-L1 cells

In order to evaluate the effects of TP-D on TG levels, the differentiated 3T3-L1 cells were treated with various concentrations of TP-D (125, 250, 500 and 1,000 g/ml). Glucose consumption induced cell differentiation and the accumulation of TGs. As noted in our previous experiment descrbied above, glucose uptake decreased when the cells were treated with TP-D (11). Therefore, we measured the TG levels under the same treatment conditions. The TG levels in the 3T3-L1 cells decreased significantly and in a dose-dependent manner following treatment with TP-D at 125, 250, 500 and 1,000 ng/ml for 48 h. The most significant decrease in the TG levels was observed at the dose of 500 and 1,000 ng/ml. Therefore, all further experiments were performed after 48 h of TP-D treatment (at 500 or 1,000 ng/ml; Fig. 2B).

### Effects of TP-D on the expression of lipogenic and adipogenic genes during the differentiation of 3T3-L1 cells

**Figure 3:**
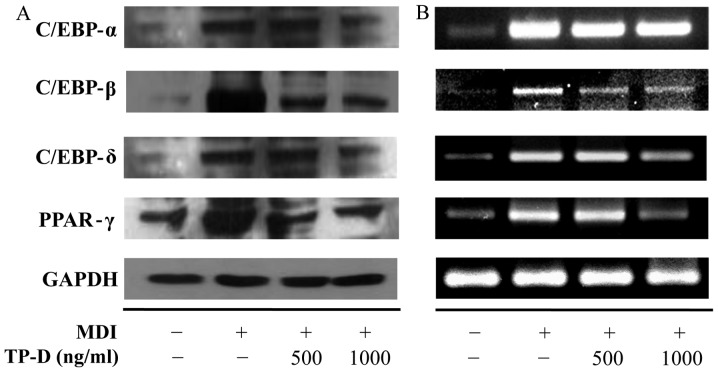
Expression levels of CCAAT/enhancer-binding proteins (C/EBPs) and peroxisome proliferator-activated receptor (PPAR)-γ in differentiated 3T3-L1 adipocytes following treatment with the tuna-derived peptide, D-I-V-D-K-I-E-I (TP-D). Cells were treated with TP-D (125, 250, 500 or 1,000 ng/ml) for 48 h. (A) Protein expression levels were measured by western blot analysis. (B) cDNA was subjected to RT-PCR, and mRNA levels were analyzed by electrophoresis on a 1% (w/v) agarose gel and staining with RedSafe nucleic acid staining solution.

**Figure 4:**
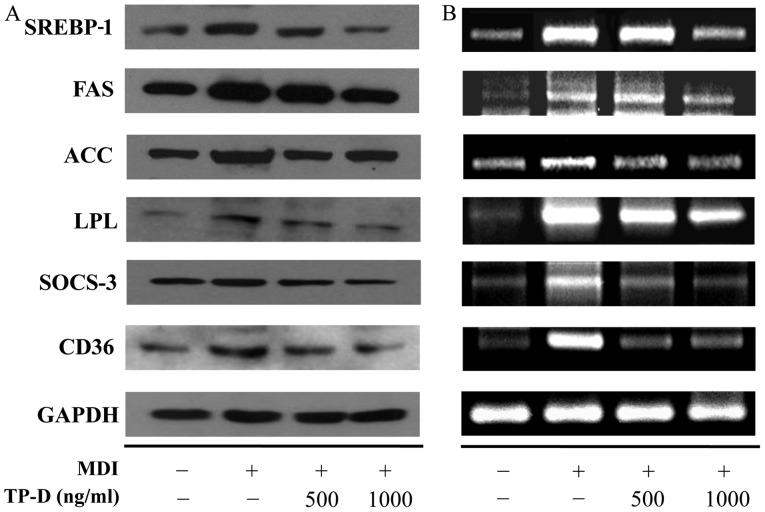
Expression levels of sterol regulatory element binding protein-1 (SREBP-1), fatty acid synthase (FAS), acetyl-CoA carboxylase (ACC), lipoprotein lipase (LPL), suppressor of cytokine signaling-3 (SOCS-3) and CD36 in differentiated 3T3-L1 adipocytes following treatment with the tuna-derived peptide, D-I-V-D-K-I-E-I (TP-D). Cells were treated with TP-D (125, 250, 500 or 1,000 ng/ml) for 48 h. (A) Protein expression levels were measured by western blot analysis. (B) cDNA was subjected to RT-PCR and mRNA expression was analyzed by electrophoresis on a 1% (w/v) agarose gel and staining with RedSafe nucleic acid staining solution.

**Figure 5:**
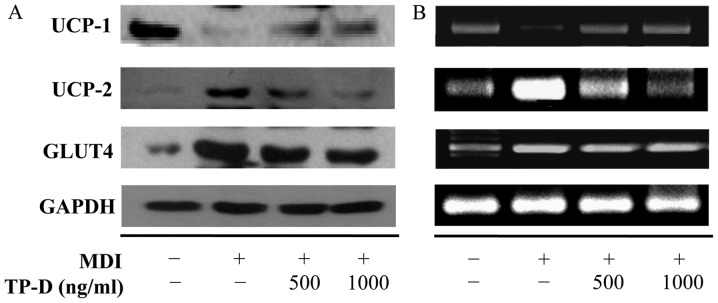
Expression levels of uncoupling protein (UCP)-1, UCP-2 and glucose transporter type 4 (GLUT4) in differentiated 3T3-L1 adipocytes following treatment with the tuna-derived peptide, D-I-V-D-K-I-E-I (TP-D). Cells were treated with TP-D (125, 250, 500 or 1,000 ng/ml) for 48 h. (A) Protein expression levels were measured by western blot analysis. (B) cDNA was subjected to RT-PCR, and mRNA expression was analyzed by electrophoresis on a 1% (w/v) agarose gel and staining with RedSafe nucleic acid staining solution.

C/EBP-β and -δ were overexpressed during the initial differentiation of 3T3-L1 cells, which was triggered by adipogenic inducers secreted by pre-adipocytes. This induced the expression of C/EBP-α and PPAR-γ, transcription factors that are important in differentiation (11). Western blot analysis and RT-PCR were used to measure the protein and mRNA expression levels, respectively. TP-D inhibited adipocyte formation by downregulating the expression of C/EBPs and PPAR-γ (Fig. 3). Liver X receptors (LXRs) are major nuclear receptors involved in the regulation of lipid synthesis. They form LXR/retinoid X receptor (RXR) heterodimers with RXR receptors and induce the activation of SREBP-1 (12–14), triggering lipid synthesis. Activated SREBP-1 then upregulates ACC, FAS, FABP and LPL. Adipogenesis and lipogenesis are thus accelerated, leading to the accumulatin of lipids (15). In addition, LXRs mediate the SREBP-1 pathway through the activation of the uptake transporter, CD36 (16). SOCS-3 is overexpressed during insulin resistance, thereby inducing SREBP-1 epxression, which is involved in the homeostatic regulation of lipid levels (17,18). Thus, in this study, we measured the expression levels of ACC, FAS, LPL, FABP, SREBP-1, SOCS-3 and CD36 by western blot analysis and RT-PCR. The expression of levels of all these factors was lower in the TP-D-treated group compared with the MDI-treated group (Fig. 4). UCPs are mitochondrial membrane transporters of brown adipose tissue. UCP-1 has a heat release function (lipids are oxidized upon the upregulation of UCP-1). UCP-2 is distributed in white adipose tissue and its expression increases as lipid cells accumulate (19). In this study, treatment with TP-D increased UCP-1 expression and decreased UCP-2 expression levels (Fig. 5). GLUT4 is involved in the active transport of glucose and its expression is increased by C/EBPs (20). In this study, treatment with TP-D decreased GLUT4 expression (Fig. 5). The inhibition of GLUT4 plays a role in insulin sensitivity by selectively inhibiting the activation of C/EBP-α by PPAR-γ.

### Effect of TP-D on the expression of Wnt-10b and the Wnt signaling pathway during 3T3-L1 differentiation

**Figure 6:**
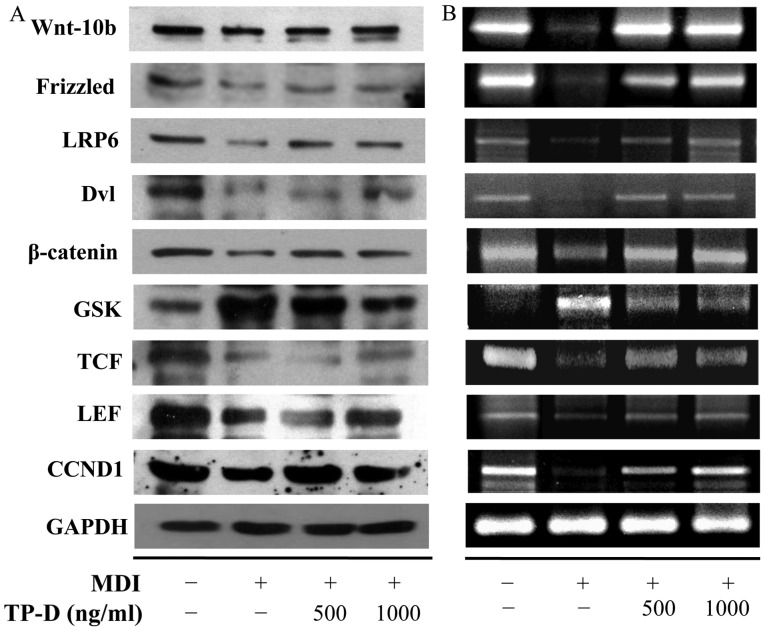
Expression levels of Wnt signaling pathway-related proteins in differentiated 3T3-L1 adipocytes following treatment with the tuna-derived peptide, D-I-V-D-K-I-E-I (TP-D). Cells were treated with TP-D (125, 250, 500 or 1,000 ng/ml) for 48 h. (A) Protein expression levels were measured by western blot analysis. (B) cDNA was subjected to RT-PCR and mRNA expression was analyzed by electrophoresis on a 1% (w/v) agarose gel and staining with RedSafe Nucleic Acid staining solution. LRP6, lipoprotein receptor-related protein-6; Dvl, dishevelled; GSK, glycogen synthase kinase; TCF, T cell factor; LEF, lymphoid enhancer-binding factor; CCND1, cyclin D1.

β-catenin, a member of the canonical Wnt signaling pathway, is known to an anti-adipogenic regulator by inhibiting the expression of C/EBP-α and PPAR-γ, which are play a major role in lipid formation (10,21). Low-density LRP6 combines with Wnt and Frizzled to bind to β-catenin, and the activation of Wnt-10b/LRP6/Frizzled induces the upregulation of Dvl (22). This occurs during the degradation of GSK-3β and catenin stabilization (23). The accumulation of catenin in the cytoplasm coincides with shuttling of the transcription factor, TCF/LEF, into the nucleus and the induction of CCND1. CCND1 inhibits C/EBP-α and PPAR-γ through a mechanism involving direct interaction with PPAR (24). In this study, we observed changes in Wnt signaling-related protein and mRNA expression by western blot analysis and RT-PCR. As shown in Fig. 6, the CCND1 and β-catenin/TCF/LEF levels increased upon the activation of Wnt-10b/LRP6/Frizzled in the cells treated with TP-D, and the GSK-3β levels decreased. These results indicated that adipogenesis and lipogenesis were decreased by the inhibition of PPAR through the upregulation of CCND1.

### Effect of TP-D on the total and HMW adiponectin levels during 3T3-L1 differentiation

**Figure 7:**
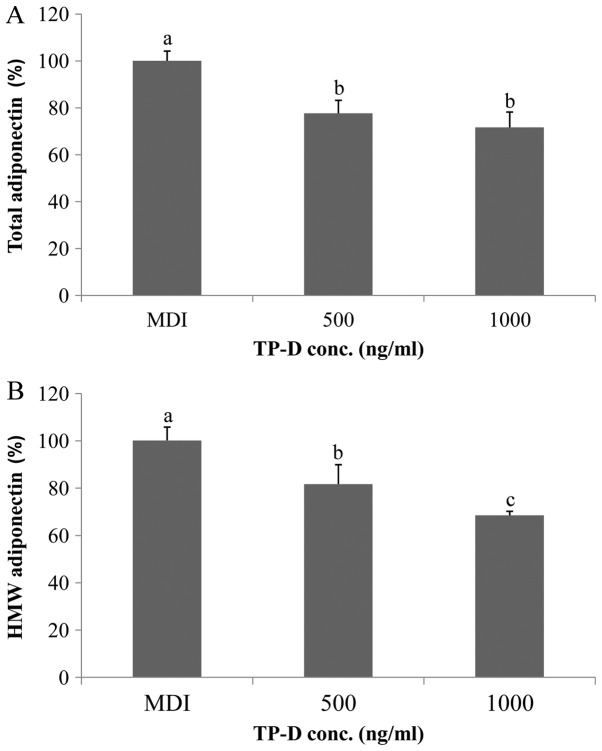
Total and high-molecular weight (HMW) adiponectin levels in differentiated 3T3-L1 adipocytes. Following differentiation, 3T3-L1 cells were treated with the tuna-derived peptide, D-I-V-D-K-I-E-I (TP-D) (500 or 1,000 ng/ml), for 48 h. The absorbances of the cell lysates were measured at 492 nm after ELISA detecting (A) total adiponectin and (B) HMW (high-molecular weight) adiponectin levels. Values represent the means ± SD; P<0.05 as shown by ANOVA. Bars labeled with different letters indicate significant differences according to Duncan’s multiple range test. conc., concentration.

In general, adiponectin forms multimers (25). HMW and total adiponectin are markers of the key transcriptional regulator, PPAR-γ (26). In this study, we examined whether TP-D affects the total and/or HMW adiponectin levels. As shown in Fig. 7, the levels of total and HMW adiponectin decreased upon treatment with TP-D, in a dose-dependent manner, indicating that PPAR-γ synthesis was inhibited.

## Discussion

Obesity is the state in which excess body fat accumulates, and it is directly caused by an increase in the number and size of abnormal adipocytes. Various attempts to prevent and manage obesity have been reported (27). The proliferation of adipocytes is closely associated with adipogenesis and lipogenesis, the processes through which pre-adipocytes differentiate into adipocytes (28). Several transcription factors regulate this process directly or indirectly; of these, PPAR-γ and C/EBPs are important factors in controlling adipogenesis during the early stages of differentiation. Therefore, it is important to control these activities during early adipogenesis in order to prevent obesity.

Recently, it has been suggested that food derived from biologically active substances can prevent obesity. Tuna is a high-protein food with anticancer and anti-atherosclerotic effects, and is known to reduce blood cholesterol levels. Boiled tuna extract contains carnosine and taurine, collagen-derived proteins, and a number of free amino acids (29). However, boiled tuna extract is typically considered a byproduct, and few studies on the material have been published to date (30,31). In this study, we evaluated the anti-obesity effects of a peptide of boiled tuna extract (sequence D-I-V-D-K-I-E-I; termed TP-D) in 3T3-L1 pre-adipocytes. We found that treatment of the cells with the peptide inhibited differentiation and decreased glucose uptake and TG levels.

During the differentiation of 3T3-L1 pre-adipocytes into adipocytes, the consumption of glucose increases and TGs accumulate. Upon the induction of the expression of PPAR and C/EBP, inducers of early differentiation, SREBP-1 is activated via SOCS-3. SREBP-1 is directly involved in lipogenesis and adipogenesis. During PPAR expression, LXR (a major nuclear receptor involved in the regulation of lipogenensis) is activated by oxysterol, a process that is also involved in SREBP expression and that may also mediate SREBP-independent lipogenesis by activating fatty acid translocase (FAT)/CD36) (12–16), an absorption carrier. Apart from the traditional adipogenic pathway, the inhibition of lipogenesis through the Wnt pathway is an anti-obesity effect. During the formation of adipocytes, the canonical Wnt signaling pathway is activated in pre-adipocytes; however, its activity decreases as cells differentiate (32). The main component of the canonical Wnt signaling pathway is β-catenin, which has a variety of functions during the growth and differentiation of several cell types (33). β-catenin activates LEF/TCF transcription factors and, in the absence of the Wnt signal transmission, is decomposed by the AXIN/GSK-3β/APC complex. The activation of the Wnt/β-catenin pathway through the inhibition of PPAR-γ is essential to the inhibition of the differentiation of pre-adipocytes. CCND1 is the direct target of the β-catenin/LEF complex and binds to the complex (34). CCND1 also inhibits PPAR-γ activation through ligand binding; the mechanisms involve direct interaction with PPAR-γ. Thus, as the inhibition of lipogenic and adipogenic pathways reduces obesity, it is crucial to inhibit C/EBPs and PPAR-γ and activate the Wnt/β-catenin pathway. In the present study, we found that a peptide derived from boiled tuna inhibited C/EBPs and PPAR-γ expression and activated the Wnt/β-catenin pathway; as a result, the process through which pre-adipocytes differentiate into fat globule cells was also inhibited.

In conclusion, the findings of our present study indicate that the tuna peptide, TP-D, may prove to be an effective strategy with which to reduce glucose uptake and TG levels and to prevent the adipocyte differentiation of 3T3-L1 cells.
